# Integrated Metabolomics and Proteomics Analysis Revealed Second Messenger System Disturbance in Hippocampus of Chronic Social Defeat Stress Rat

**DOI:** 10.3389/fnins.2019.00247

**Published:** 2019-03-22

**Authors:** Li-Ning Yang, Jun-Cai Pu, Lan-Xiang Liu, Guo-Wei Wang, Xin-Yu Zhou, Yu-Qing Zhang, Yi-Yun Liu, Peng Xie

**Affiliations:** ^1^Department of Neurology, The First Affiliated Hospital of Chongqing Medical University, Chongqing, China; ^2^Institute of Neuroscience and the Collaborative Innovation Center for Brain Science, Chongqing Medical University, Chongqing, China; ^3^School of Clinical Medicine, Ningxia Medical University, Yinchuan, China; ^4^Department of Psychiatry, The First Affiliated Hospital of Chongqing Medical University, Chongqing, China; ^5^Department of Neurology, The Second Affiliated Hospital of Chongqing Medical University, Chongqing, China

**Keywords:** depression, social defeat, proteomics, metabolomics, second-messenger

## Abstract

Depression is a common and disabling mental disorder characterized by high disability and mortality, but its physiopathology remains unclear. In this study, we combined a non-targeted gas chromatography-mass spectrometry (GC-MS)-based metabolomic approach and isobaric tags for relative and absolute quantitation (iTRAQ)-based proteomic analysis to elucidate metabolite and protein alterations in the hippocampus of rat after chronic social defeat stress (CSDS), an extensively used animal model of depression. Ingenuity pathway analysis (IPA) was conducted to integrate underlying relationships among differentially expressed metabolites and proteins. Twenty-five significantly different expressed metabolites and 234 differentially expressed proteins were identified between CSDS and control groups. IPA canonical pathways and network analyses revealed that intracellular second messenger/signal transduction cascades were most significantly altered in the hippocampus of CSDS rats, including cyclic adenosine monophosphate (cAMP), phosphoinositol, tyrosine kinase, and arachidonic acid systems. These results provide a better understanding of biological mechanisms underlying depression, and may help identify potential targets for novel antidepressants.

## Introduction

Depression is one of the most prevalent psychiatric and disability diseases conditions in the world today, but its physiopathology remains poorly elucidated (Health TNIoM, 2016; World Health Organization [WHO], 2017). Among various animal models, social defeat stress is one of the most widely used for investigating possible causes of and treatments for depression (Becker et al., 2008; Ota et al., 2014). Chronic social stress requires learned social defeat for a long period, usually combined with repeated exposure to sensory stimuli from aggressive rodents (Denmark et al., 2010). The repeated exposure of rodents to social defeat causes a solid depression-like behavior marked by anhedonia, body weight alteration, and altered protein expression in various brain areas (Vyas et al., 2002; Radley and Morrison, 2005; Denmark et al., 2010).

Hippocampus is a key brain area for emotion and motivation generation and regulation, and plays a critical influence in the etiology of depression (Campbell and Macqueen, 2004; McEwen et al., 2012). Neuropathological studies of hippocampus showed atrophy of hippocampal neurons (Sousa et al., 2000; Harrison, 2002; Lucassen et al., 2006; McEwen, 2010). However, how these changes in hippocampus lead to depression still unclear (Christoffel et al., 2011). Revealing these molecular events is essential for understanding the pathogenesis of depression.

In recent decades, omics technologies have become a powerful tool to uncover the physiopathology of neuropsychiatric disorders (Zheng et al., 2013a, 2016a; Zhou et al., 2015). Metabolomics can quantify low-molecular-weight metabolites in specific biological sample, and is widely used to capture disease-specific metabolite signatures (Lindon and Nicholson, 2008; Koike et al., 2014; Proitsi et al., 2015). Proteomics – the analysis of protein expression in biological samples – can provide insights into the pathophysiological mechanisms of several disease states (Taurines et al., 2011). Previously, our team completed a series of research on depression in clinical patients and animal models using a single omics technology (Zheng et al., 2013b, 2016b; Chen et al., 2015; Liu et al., 2016; Wang et al., 2016; Zhou et al., 2018). Recently, researchers have started to use combinatorial omics approaches to study the physiopathology of various diseases, including cancer, cardiovascular disease, and psychiatric disorders (Mayr et al., 2008; Ma et al., 2012; Wesseling et al., 2013). In those studies, metabolomics informed functional interpretations of proteomic results, and proteomics helped to a better understanding of metabolomics results by emphasizing the involvement of specific enzymatic and/or enzymatic pathways. With respect to depression, significantly perturbed energy metabolism in the cerebellum and dysfunction of amino acid metabolism and lipid metabolism in the hippocampus of chronically stressed rats has been observed, and disturbance of phospholipid metabolism in the plasma of MDD patients has been found in previous studies using integrated analysis of proteomic and metabolomic profiles (Shao et al., 2015; Gui et al., 2018; Zhang et al., 2018). Hence, combining proteomic analysis with metabolic profiling is promising and may help to elucidate complete biological mechanisms (Cavill et al., 2016).

The purpose of this research was to elucidate metabolite and protein alterations induced by depression in rat hippocampus after CSDS. We combined a non-targeted GC-MS-based metabolomic approaches with iTRAQ-based proteomic analysis. Furthermore, IPA was conducted to integrate potential relationships among altered metabolites and proteins to better understand the physiopathology of depression.

## Materials and Methods

### Animals and Ethics Statement

This research (including stress and behavioral tests) was conducted in accordance with recommendations of the Guide for the Care and Use of Laboratory Animals, and approved by the Ethics Committee of Chongqing Medical University. All procedures contributing to this work comply with the ethical standards of the relevant national and institutional committees on animal experimentation. As described in our previous study, thirty-five male SD rats, 250–300 g, were kept in a separate cage as experimental intruder or control animals (Liu et al., 2018). Male LE rats, 380–450 g, were housed in pairs with ligatured female LEs served as resident rats. All animals lived under a 12-h day–and-night regime (lights on at 19:00) in controlled environmental conditions (21 ± 1°C, 55 ± 5% relative humidity) to ensure that all manipulations and tests were performed during the active phase (subjective night) of rats. Food and water were available *ad libitum*. Before starting the experimental procedures, all animals were adapted to light regime, controlled environmental conditions, as well as handling and presence of the experimenter in the room for 10 days.

Using measurements of body weight, sucrose preference, and activity, outliers at baseline were removed, leaving twenty-eight SD rats that were body weight matched and randomly divided into the CSDS group or CON group.

### Experimental Procedures

#### Social-Defeat Procedure

**Figure 1:**
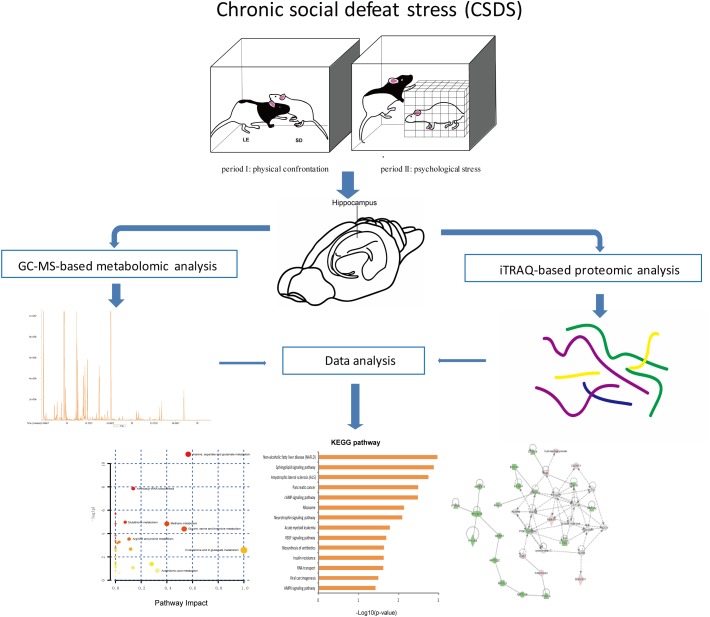
Experimental flowchart.

The social defeat pattern included two consecutive periods (a total 60 min) in LE rats’ home cages. During period I (5 min), rats were allowed physical confrontation. In period I, when the interaction became too strong or once the SD rat surrendered or acquired a supine position over 5 seconds, the SD rat was transported to a small wire mesh protective cage (10 cm × 10 cm × 15 cm) within the LE’s home and spend the remaining 60 min (period II). Hence, the wire mesh cage allowed for comprehensive visual, olfactory, as well as auditory exposure to LE rat without direct physical contact. CON group rats were placed in the empty LE rats’ home cages for 60 min. The 60-min social defeat exposure was repeated once daily for 5 days during weeks 1 and 3, and once daily for 3 days during week 2. The overall experimental procedure is shown in Figure 1.

#### Behavioral Tests

Weight measurement and SPT were conducted at baseline and weekly during for 3 weeks. In addition, during the whole experiment, a series of behavioral tests were carried out in a soundproof room between 09:00 and 15:00 with a red light (1–2 lx), as mentioned in earlier publication (Liu et al., 2018). LAT was conducted only at baseline to test the activity of rats prior to stress. OFT, EPM, and FST were conducted after the CSDS procedure. Prior to LAT, FST, OFT, and EPM testing, each rat was individually transported to the testing room and adapted for 6–7 min. The testing order was alternated between CSDS and CON groups.

#### Sample Preparation

After a short period of anesthesia, two hippocampi were quickly dissected and separately immersed into liquid nitrogen and maintained at -80°C before analysis.

#### Metabolomic Analysis

Details of GC-MS approaches and procedures for metabolomic analysis were provided in our previous study (Liu et al., 2018). Metabolomic analysis was performed using SIMCA software (version 14.0) for principal component analysis (PCA) and OPLS-DA. The criterion for identifying significantly different metabolites was variable influence of projection (VIP) >1 and *p-values* <0.05. Heatmap and unsupervised hierarchical clustering analysis of differentially expressed metabolites were constructed using R (version 3.4.0). The MetaboAnalyst online tool (version 4.0) was performed to explore biological patterns, functions, and pathways of identified differentially expressed metabolites.

#### Proteomic Analysis

As we previously described, iTRAQ-based proteomic measurements were made using an iTRAQ Reagent-8plex Multiplex Kit (Zhang et al., 2018). Two pooled samples were obtained corresponding to 9 or 8 rats in each group (CSDS group: *n* = 9; CON group: *n* = 8), then each pooled sample was divided into three equal parts as replicates. Subsequently, peptides were mixed and fractionated by strong cation exchange chromatography, liquid chromatography–tandem mass spectrometry analysis, analysis by Q Exactive, and sequence database searching and analysis. Compared with the CON group, final proteins considered to be differentially expressed and chosen for further analysis by *p-values* <0.05 and 1.2-fold changes (>1.20 or <0.83) for proteomic analysis (Ren et al., 2013). Heatmap and hierarchical clustering analysis of feature proteins between two sample sets were constructed using R (version 3.4.0). The DAVID online tool (version 6.8) was used to annotate the function of differentially expressed proteins. The STRING online tool (version 9.0) was used to obtain differentially expressed protein interaction data.

#### Statistical Analysis

The results of behavioral tests including body weight, SPT, OFT, EPM, and FST are presented with mean ± standard deviation and calculated by IBM SPSS (version 21.0). For all analyses, *p-values* <0.05 were considered to have statistical significance.

#### Integrated Analysis of Metabolomics and Proteomics

Ingenuity pathway analysis (IPA Ingenuity Systems, Redwood City, CA, United States) software was used to analyze and integrative metabolites and proteins data (including canonical pathway and molecular interaction network). The network score was based on a hypergeometric distribution and calculated using the right-tail Fisher exact test.

## Results

After screening, there were 28 rats (20 CSDS and 8 CON) for subsequent experiments. No significant differences in LAT, body weight, or SPT were found between CSDS and CON groups at baseline. After 3 weeks of social-defeat exposure, the 20 CSDS rats were divided into susceptible and resilient subgroups according to their SPT. In this research, only susceptible rats (9 CSDS) were used for further analysis, with no significant differences in baseline body weight (281.94g vs. 289.06g, *p* = 0.392), SPT (84.01% ± 9.83% vs. 85.02% ± 7.70%, *p* = 0.819), or LAT (1544.20 cm vs. 1624.64 cm, *p* = 0.700) between CSDS (*n* = 9) and CON (*n* = 8) groups (Supplementary Figure S1).

The results of behavioral tests were reported in our previous publication (Liu et al., 2018). Briefly, the CSDS group gained less body weight than the CON group after 1 week (293.38g vs. 314.99g, *t* = -2.70, *p* < 0.05), 2 weeks (305.04g vs. 335.83g, *t* = -3.52, *p* < 0.05), and 3 weeks (324.83g vs. 356.28g, *t* = -3.12, *p* < 0.05) (Supplementary Figure S1A). The sucrose preference of CSDS group was lower than that of CON group (85.98% vs. 91.41%, *p* < 0.05; Supplementary Figure S1B). In FST, immobility time of CSDS group was longer than that of CON group (130.63 vs. 112.39, *p* < 0.05; Supplementary Figure S1C), indicating aggravated despair behavior. In OFT, total distance (2804.82 vs. 3249.95, *t* = -1.16, *p* > 0.05; Supplementary Figure S1D), time spent in the inner squares, and number of rearing behaviors (8.94 vs. 9.91, *t* = -0.26, *p* > 0.05; 13.78 vs. 14.25, *t* = -0.14, *p* > 0.05; Supplementary Figures S1E,F) did not differ between the two groups, indicating no anxiety-like behavior in this model. In EPM, time spent in open arms and frequency (67.78 vs. 40.81, *z* = -0.55, *p* > 0.05; 3.44 vs. 2.50, *t* = 0.80, *p* > 0.05; Supplementary Figures S1G,I), as well as time spent in closed arms and frequency (120.06 vs. 158.44, *t* = -0.27, *p* > 0.05; 11.00 vs. 11.63, *t* = -1.34, *p* > 0.05; Supplementary Figures S1H,J) did not differ between the two groups.

### GC-MS-Based Metabolomic Analyses

**Figure 2:**
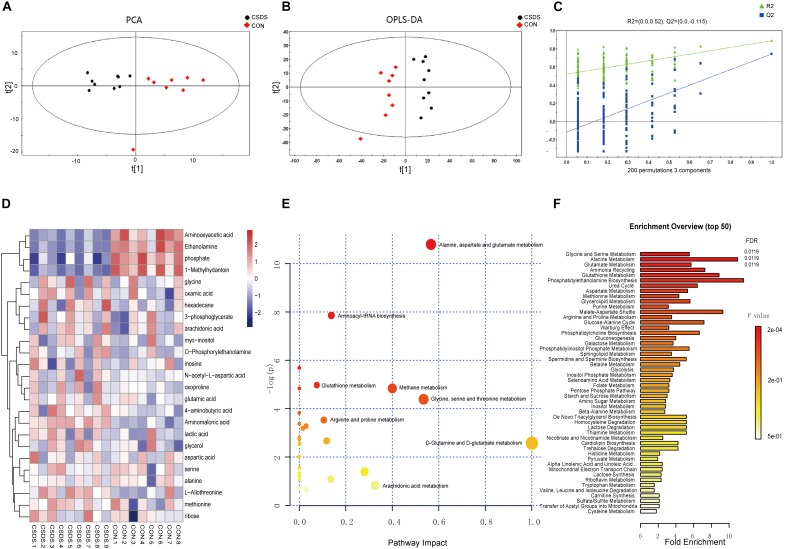
Metabolomic analysis of hippocampi from CSDS and CON group rats. **(A)** Principal component analysis (PCA) scores plot of CSDS group rats (black dots) and CON group rats (red diamonds). **(B)** Orthogonal partial least-squares discriminant analysis (OPLS-DA) scores of CSDS group rats (black dots) and CON group rats (red diamonds). **(C)** A 200-iteration permutation test showing the original values (top right) of R^2^ (green triangles) and Q^2^ (blue squares) was significantly higher than corresponding permuted values (bottom left), demonstrating the OPLS-DA model was not-overfitted. **(D)** Heatmap of 25 differentially expressed metabolites in the CSDS model: rows, metabolites; columns, samples. **(E)** Overview of pathway analysis based on metabolite alterations. **(F)** Overview of enrichment analysis based on metabolite alterations.

Using GC-MS metabolomic profiling of hippocampus from CSDS and CON groups, 413 metabolites were identified and used for subsequent multi-variate analysis. PCA score indicated clear segregation between CSDS group and CON group (*R^*2*^X* = 0.431, *Q^*2*^* = 0.147; Figure 2A). In addition, the resulting OPLS-DA scores plot clearly distinguished CSDS group from CON group (*R^*2*^X* = 0.896, *R^*2*^Y* = 0.890, *Q^*2*^* = 0.762; Figure 2B). The results of permutation tests showed no overfitting in the OPLS-DA model (*R^*2*^* = 0.52, *Q^*2*^* = -0.115; Figure 2C). Based on the OPLS-DA model, 25 differentially expressed metabolites were identified between CSDS and CON groups using criteria of VIP > 1 and *p-values* < 0.05 (Supplementary Table S1). Heatmap visualization of 25 differential metabolomics data is displayed in Figure 2D. Overviews of pathway and enrichment analyses based on metabolite alterations are displayed in Figure 2E,F.

### iTRAQ-Based Proteomic Analyses

**Figure 3:**
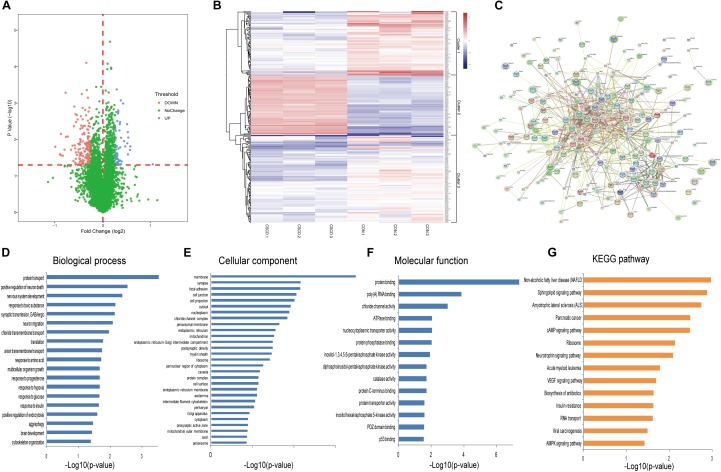
Proteomic analysis of hippocampi from CSDS and CON groups rats. **(A)** Volcano plot of all proteins. **(B)** Heatmap of 234 differentially expressed proteins in the CSDS model: rows, proteins; columns, samples. **(C)** Protein-Protein interaction (PPI) network of 196 differently expressed proteins which was performed with STRING. Nodes represent proteins. Edges represent protein-protein associations. **(D–F)** Distribution of altered proteins for biological processes, cellular components, and molecular functions. Only GO terms that were significantly overrepresented (*p* < 0.05) are shown. **(G)** Significantly enriched pathways identified by KEGG pathway analysis (*p* < 0.05).

Using iTRAQ-based proteomic profiling of hippocampus from CSDS and CON groups, 4950 proteins were detected with at least one unique peptide and a 1% FDR. A volcano plot of all proteins is displayed in Figure 3A. Based on the criteria mentioned above, 234 differentially expressed proteins were identified and chosen for further analysis between CSDS group and CON group (Supplementary Table S2). Among them, 66 proteins were increased and 168 were decreased expression. Heatmap visualization of 234 differentially expressed proteins is displayed in Figure 3B. As shown in the Figure 3B, CSDS group and CON group were clearly separated, and three replicates of each group showed good reproducibility. According to hierarchical clustering analysis, proteins were resulted into three clusters. The proteins in cluster 1 were most associated with positive regulation of neuron death, while those in cluster 2 were most associated with translation, and those in cluster 3 were most associated with protein transport. Gene Ontology (GO) analysis annotated 221 proteins and then resulted them into 19 significant GO terms for biological processes (Figure 3D), 29 for cellular components (Figure 3E), and 14 for molecular function (Figure 3F). A total of 196 altered proteins were annotated by STRING. The Protein-Protein interaction (PPI) network of these proteins is showed in Figure 3C. The PPI network analysis revealed that MAP3K5, PIK3R2, RAC1, PIP4K2C, and PPP2R2B participated in various biological progress such as single-organism cellular process or single-organism transport, and MAP3K5, PIK3R2, RAC1, PIP4K2C, and BAD participated in neurotrophin signaling pathway, which may play a critical influence in the pathogenesis of depression. The significantly different canonical KEGG pathways are displayed in Figure 3G.

### Integrated Pathway and Network Analyses

**Figure 4:**
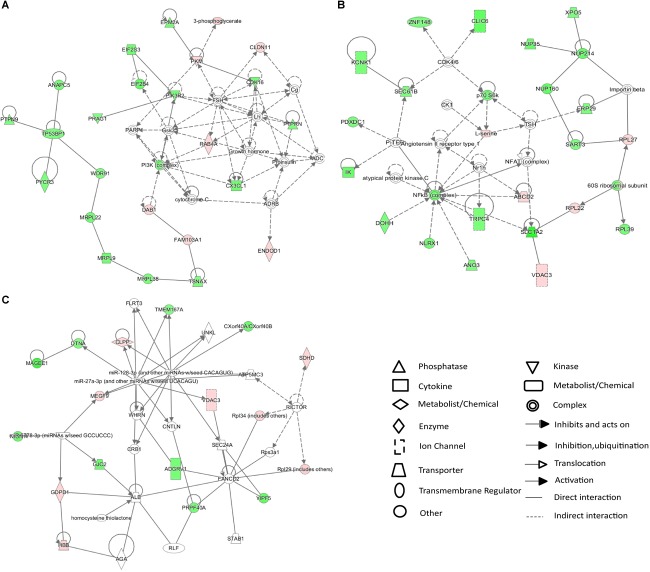
IPA network analysis of differentially expressed proteins and metabolites in the CSDS model group. **(A–C)** Top-ranked enriched networks based on changed products. Red, significantly increased; green, significantly decreased.

The 25 differentially expressed metabolites and 234 differentially expressed proteins were submitted to IPA for integration analysis. According to canonical pathway analysis, the top five significantly different canonical pathways were Rac signaling, ceramide signaling, folate transformation I, S-methyl-5-thio-a-D-ribose 1-phosphate degradation, and regulation of eIF4 and p70S6K signaling (Supplementary Table S3). In the network function analysis, “cellular movement, cell death and survival, molecular transport” changed most significantly, with a score of 43 (Figure 4A). The “molecular transport, RNA trafficking, protein trafficking” and “auditory disease, hereditary disorder, neurological disease” networks were also identified as top-ranked enriched networks based on changed products, with scores of 41 and 27 respectively (Figure 4B,C). Molecular and cellular functions related to submitted metabolites and proteins were molecular transport, amino acid metabolism, small molecule biochemistry, cellular assembly and organization, and cellular function and maintenance.

## Discussion

**Figure 5:**
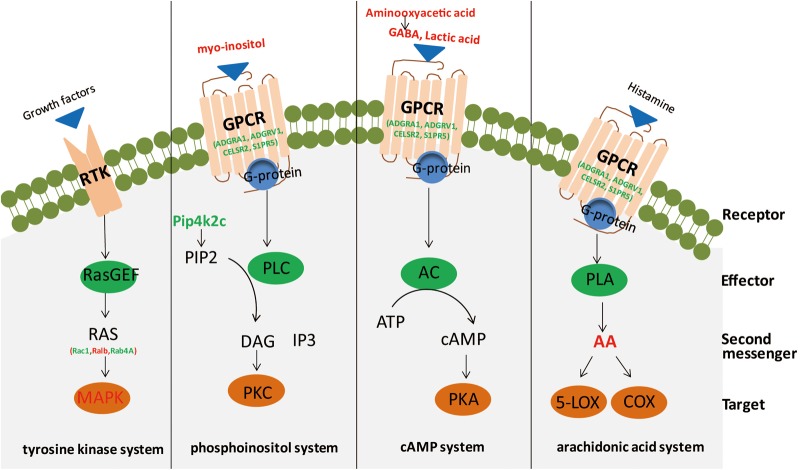
Second messengers disseminate information received from cell-surface receptors. A simplistic diagram of second messenger systems, including tyrosine kinase, phosphoinositol, cyclic adenosine monophosphate (cAMP), and arachidonic acid (AA) systems. In the tyrosine kinase system, binding of growth factors to a receptor tyrosine kinase (RTK; the receptor) can activate RasGEF (the effector) to produce RAS (the second messenger) to activate mitogen-activated protein kinase (MAPK; the target). For the phosphoinositol system, binding of ligands to a GPCR (receptor) activates phospholipase C (PLC; effector), to generate 2 s messengers, DAG and IP3, which activate protein kinase C (PKC; target) and release calcium from intracellular stores. Red, significantly increased; green, significantly decreased. In the cAMP system, binding of agonists to a GPCR (receptor) can activate adenylyl cyclase (AC; effector) to produce cAMP (second messenger) to activate protein kinase A (PKA; target). In the AA system, binding of ligands to a GPCR (receptor) can activate phospholipase A (PLA; effector) to generate AA (second messenger), which activates targets cyclooxygenase (COX) or 5-lipoxygenase (5-LOX).

In the current research, we conducted an integrated analysis of GC–MS-based metabolomics and iTRAQ-based proteomics to investigate molecular changes in the hippocampus of rats induced by CSDS. Twenty-five significantly different metabolites and 234 differentially expressed proteins were identified between CSDS and control groups. IPA canonical pathway and network analyses of integrated metabolites and proteins revealed that biomolecular changes in the hippocampus of CSDS rats are mainly related to intracellular second messenger/signal transduction cascades (Figure 5).

Among the 25 differentially expressed metabolites, 21 in the hippocampus were also considered as potential biomarkers of depression in earlier researches, but most of these in chronic unpredictable mild stress model (Kim et al., 2014; Du et al., 2016; Liu et al., 2016; Zhang et al., 2018). The remaining metabolites (myo-inositol, aminomalonic acid, ribose, and oxamic acid) are identified as potential biomarkers for the first time. Consistent with a previous study, most are primarily involved in amino acid metabolism, carbohydrate metabolism, and lipid metabolism (Zhang et al., 2018). GO enrichment analysis of the 234 differentially expressed proteins revealed that most proteins are associated with protein transport, positive regulation of neuron death, nervous system development, response to toxic substances and synaptic transmission, and GABAergic. KEGG pathway analysis based on differentially expressed proteins revealed that sphingolipid signaling, cAMP signaling, and neurotrophin signaling, which have previously been associated with depression, are the main signaling pathways activated in the hippocampus of CSDS rats (Gould and Manji, 2002; Niciu et al., 2013).

Ingenuity pathway analysis canonical pathway and network analyses of integrated metabolites and proteins revealed that intracellular second messenger/signal transduction cascades were the most significantly altered in the hippocampus of CSDS rats. Indeed, the results of IPA canonical analyses showed six of the top ten pathways are related to second messenger signaling, including Rac signaling, ceramide signaling, regulation of eIF4 and p70S6K signaling, mTOR signaling, nerve growth factor signaling, and PI3K/AKT signaling (Niciu et al., 2013; Newton et al., 2016). The results of IPA network analyses revealed that the network “cellular movement, cell death and survival, molecular transport” was the most altered between CSDS and CON groups. Our findings are consistent with previous studies using animal models of depression or clinical patients of MDD, which revealed imbalance in second messenger/signal transduction cascades (Niciu et al., 2013). Several researchers have examined the critical influence of second messenger/signal transduction cascades in the physiopathology and treatment of depression, e.g., cAMP/PKA/CREB, neurotrophin-mediated (MAPK and others), p11, Wnt/Fz/Dvl/GSK3β, NFκB/Δfobs, mTOR, and eEF2K/CAMKIII, resulting in the second-messenger imbalance hypothesis (Wachtel, 1989, 1990; Niciu et al., 2013). In addition, previous studies found that more direct targeting of second messenger/signal transduction intermediate products may offer a faster and more solid effects than currently available antidepressants (Li et al., 2010; Autry et al., 2011; Monteggia et al., 2013).

Several distinct secondary messenger systems, such as cAMP, phosphoinositol, tyrosine kinase, and AA systems, are reportedly associated with depression (Odagaki et al., 2001; Gould and Manji, 2002; Skelin et al., 2011; Niciu et al., 2013; Fujita et al., 2017). The cAMP second messenger system plays a pivotal role in neuroplasticity and depression; in addition, studies have shown that antidepressant treatment can alter the expression of components of this signaling pathway in rodents (Popoli et al., 2000; Fujita et al., 2017). In this research, we found that GABA, lactic acid, and aminooxyacetic acid were significantly elevated in the hippocampus. Among these three metabolites, GABA and lactic acid can participate in the cAMP signaling pathway through binding and activation of GPCRs, and aminooxyacetic acid can inhibit GABA-T activity, leading to a reduction of GABA breakdown (Wallach, 1961). Moreover, GPCRs (including ADGRA1, ADGRV1, CELSR2, and S1PR5) previously reported to be implicated in the physiopathology and pharmacology of depression were also significantly altered (Tomita et al., 2013). Whereas previous studies observed decreased activation of adenylate cyclase and PKA in depression (Gould and Manji, 2002; Akin et al., 2005), in this study, we found a significant decrease of PPP1R13B and PPP2R2B, two phosphate family proteins that counterbalance the action of PKA (Fimia and Sassone-Corsi, 2001), in the hippocampus. Unexpectedly, but consistent with earlier researches, we did not observe changes in cAMP levels (Belmaker et al., 1980; Maj et al., 1984). However, changes in receptor-mediated cAMP formation have been studied in depression (Dwivedi and Pandey, 2008). These researches indicate PKA and associated cAMP signaling molecules may act as crucial neurobiological factors in depression (Dwivedi and Pandey, 2008).

Alterations of phosphoinositol signaling in the cerebra and peripheral tissues of depressed patients have been extensively reported, and studies have found that mood stabilizers (e.g., lithium, and valproate) seem to partially repair these abnormalities (Lenox et al., 1992; Kofman and Belmaker, 1993; Yuan et al., 2001; Gould and Manji, 2002). In this study, significantly altered metabolites and proteins (including myo-inositol, PLD2, and PIP4K2C) involved in phosphoinositol second messenger systems were observed. Myo-inositol is a by-product of membrane-bound phospholipid metabolism and an important player in the phosphoinositide secondary messenger pathway (Yildiz-Yesiloglu and Ankerst, 2006). A previous study reported that elevated levels of myo-inositol could arise from disturbances in the coupling metabolism of the receptor-second messenger system complex, thus providing an important biological substrate for the onset of depression (Hemanth Kumar et al., 2012). Protein PIP4K2C may play a critical influence in the generation of phosphatidylinositol bisphosphate, which is a precursor for second messengers of phosphoinositide signaling pathways (Itoh et al., 1998; Bulley et al., 2015). Meanwhile, PLD2 is an isoenzyme of PLD, which catalyzes the hydrolysis of phosphatidylcholine to generate an important lipid second messenger, phosphatidic acid (Kolesnikov et al., 2012; Zhu et al., 2018). These results are consistent with our previous plasma proteomic and metabolomic study, which found the dysregulation of phospholipid metabolism may aggravate inflammation in the central nervous system, ultimately leading to depression (Gui et al., 2018).

Small G proteins, which function as monomeric small GTPases, form one of the two classes of G proteins. In this study, elevated levels of three small GTPases, Rac1, RALB, and RAB4A, were observed. In addition, a significantly altered protein level of MAP3K5, a member of the MAP kinase kinase kinase family and part of the mitogen-activated protein kinase pathway, was observed. These factors are reportedly involved in signal transduction as second messengers for the tyrosine kinase system. AA, a polyunsaturated fatty acid that plays an important role in regulating inflammation signals and (possibly) vulnerability to depression, participated in cellular signaling as a lipid second messenger (Janssen-Timmen et al., 1994; Lotrich et al., 2013; Newton et al., 2016).

Furthermore, there are other metabolites and proteins involved in second messenger systems, including serine, O-phosphorylethanolamine, NAA, and protein PIK3R2. Serine and O-phosphorylethanolamine are involved in sphingolipid metabolism and sphingolipid signaling. In recent studies, sphingolipids (more specifically ceramides) were proposed to act as lipid second messengers in intracellular signaling pathways and participate in the induction of apoptosis in many cells activated by GPCR or stress stimuli (Venkataraman and Futerman, 2000; Guzmán et al., 2001; Newton et al., 2016). Recent reports suggest NAA is a main format of storage and transport of acetyl-coenzyme A in the neural system, where it is proposed to act as a second messenger relaying extracellular signals to the intracellular milieu (Ariyannur et al., 2010; Pietrocola et al., 2015). A recent research demonstrated that changes in the PI3K/AKT pathway may have specific therapeutic effects on depression (Kitagishi et al., 2012). In our study, we observed decreased PIK3R2, a regulatory subunit of PI3K, which belongs to the PI3K family. PI3Ks are enzymes that play an essential influence in lipid second messengers production and numerous biological reaction transduction.

There are some limitations of this study. First, the total number of included samples is relatively small, which may restrict interpretation of our results. Second, a number of low polarity metabolites are not detected by GC–MS. Therefore, GC–MS combined with other metabolomics approaches should be considered in future metabolomic studies. Third, iTRAQ and label-free, two quantitative proteomic analysis methods, have their own superiorities. The combination of these two methods can be complementary and should be considered in future studies. Fourth, proteomic results have not been validated by targeted methods. Thus, further researches are needed to validate these findings.

## Conclusion

In summary, we applied complementary omics strategies (GC–MS-based metabolomics and iTRAQ-based proteomics) to comprehensively elucidate metabolite and protein alterations in the hippocampus of rat after CSDS. The results of this study suggest that disturbances in several different secondary messenger systems (cAMP, phosphoinositol, AA, and tyrosine kinase) are involved in the hippocampus of CSDS rodents. An imbalance of second messengers may be related to the physiopathology of depression. The results of our study contribute to a better understanding of biological mechanisms and physiopathology of depression, and may contribute to identify novel targets for antidepressant treatment.

## Author Contributions

L-NY and PX conceived and designed the study. L-NY, J-CP, L-XL, X-YZ, Y-QZ, and Y-YL collected the data. L-NY, J-CP, and L-XL analyzed the data. L-NY wrote the first draft of the manuscript. L-NY, G-WW, and PX interpreted the data and wrote the final version. All authors approved final version of the manuscript.

## Conflict of Interest Statement

The authors declare that the research was conducted in the absence of any commercial or financial relationships that could be construed as a potential conflict of interest.

## Supplementary Material

FIGURE S1 Results of Behavioral tests. **(A)** Body weight change between CSDS group (*n* = 9) and CON group (*n* = 8). **(B)** Sucrose preference changes between CSDS and CON groups. **(C)** Immobility time in the forced swim test. **(D–F)** Total distance, time spent in the inner squares, and rearing frequency in the open field test, respectively. **(G–J)** Open arms time, closed arms time, number of open arms entries, and number of closed arms entries in the elevated plus-maze, respectively. ^∗^*P* < 0.05.

TABLE S1 Identified differential metabolites in the hippocampus between the CSDS and CON groups.

TABLE S2 Identified differential proteins in hippocampus between the CSDS and CON groups.

TABLE S3 Top ten canonical pathways by IPA analysis of integrated data from proteomics and metabolomics.

## Abbreviations

AA: arachidonic acid
cAMP: cyclic adenosine monophosphate
CON: non-stress control
CSDS: chronic social defeat stress
EPM: elevated plus maze
FDR: false discovery rate
FST: forced swim test
GABA: 4-aminobutyric acid
GABA-T: 4-aminobutyrate aminotransferase
GC-MS: gas chromatography-mass spectrometry
GPCRs: G protein-coupled receptors
IPA: ingenuity pathway analysis
iTRAQ: isobaric tags for relative and absolute quantitation
LAT: locomotor activity test
LC–MS: liquid chromatography-mass spectrometry
LE: long-evans
MDD: major depressive disorder
NAA: N-acetyl-L-aspartic acid
NMR: nuclear magnetic resonance
OFT: open field test
OPLS-DA: orthogonal partial least-squares discriminant analysis
PCA: principal components analysis
PI3K: phosphoinositide-3-kinase
PKA: protein kinase A
PLD: phospholipase D
SD: sprague-dawley
SPT: sucrose preference test
VIP: variable importance on projection.
