# Supplementary figures and images for: Integrated Metabolomics and Proteomics Analysis Revealed Second Messenger System Disturbance in Hippocampus of Chronic Social Defeat Stress Rat
Source: Front Neurosci. 2019 Mar 22;13:247. doi: 10.3389/fnins.2019.00247 (PMC6448023; doi:10.3389/fnins.2019.00247)

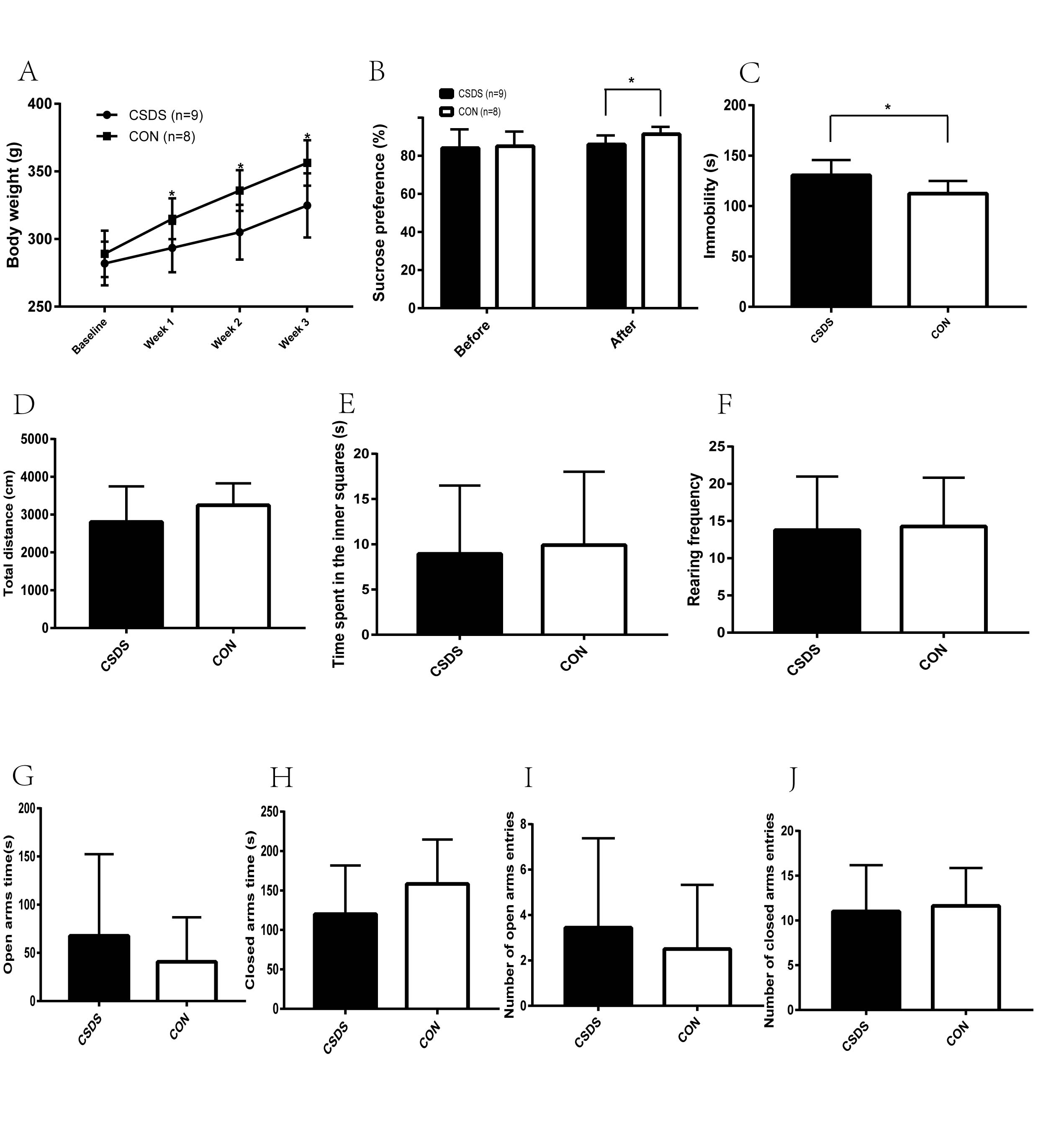

Supplement: FIGURE S1 — Results of Behavioral tests. (A) Body weight change between CSDS group (n = 9) and CON group (n = 8). (B) Sucrose preference changes between CSDS and CON groups. (C) Immobility time in the forced swim test. (D–F) Total distance, time spent in the inner squares, and rearing frequency in the open field test, respectively. (G–J) Open arms time, closed arms time, number of open arms entries, and number of closed arms entries in the elevated plus-maze, respectively. ∗P < 0.05. [file Image_1.JPEG]
