# Supplementary material for: Integrated Metabolomics and Proteomics Analysis Revealed Second Messenger System Disturbance in Hippocampus of Chronic Social Defeat Stress Rat
Source: Front Neurosci. 2019 Mar 22;13:247. doi: 10.3389/fnins.2019.00247 (PMC6448023; doi:10.3389/fnins.2019.00247)
Supplement: TABLE S1 — Identified differential metabolites in the hippocampus between the CSDS and CON groups. [file Table_1.DOCX]

**Table 1.** Identified differential metabolites in the hippocampus between the CSDS and CON groups.

| **No.** | **Metabolites** | **PubChem** | **Fold change^a^** | **p-value** | **VIP** | **Pathways** |
| --- | --- | --- | --- | --- | --- | --- |
| 1 | 1-Methylhydantoin | 69217 | 0.59 | ＜0.001 | 1.41 | Amino acid metabolism |
| 2 | 4-Aminobutyric acid | 119 | 1.42 | ＜0.001 | 1.79 | Amino acid metabolism |
| 3 | Alanine | 5950 | 1.33 | 0.042 | 2.33 | Amino acid metabolism |
| 4 | Aminomalonic acid | 100714 | / | ＜0.001 | 1.42 | Amino acid metabolism |
| 5 | Aspartic acid | 5960 | 1.31 | 0.02 | 2.22 | Amino acid metabolism |
| 6 | Glutamic acid | 33032 | 1.4 | 0.004 | 2.25 | Amino acid metabolism |
| 7 | L-Allothreonine | 99289 | 7.7 | ＜0.001 | 1.21 | Amino acid metabolism |
| 8 | Methionine | 6137 | 1.97 | 0.006 | 1.09 | Amino acid metabolism |
| 9 | N-acetyl-L-aspartic acid | 65065 | 1.34 | 0.004 | 1.4 | Amino acid metabolism |
| 10 | Oxoproline | 7405 | 1.32 | 0.004 | 4.33 | Amino acid metabolism |
| 11 | Serine | 5951 | 1.32 | 0.046 | 2.56 | Amino acid metabolism |
| 12 | Arachidonic acid | 444899 | 1.54 | 0.041 | 1.22 | Lipid metabolism |
| 13 | Ethanolamine | 700 | 0.54 | 0.01 | 1.09 | Lipid metabolism |
| 14 | Glycine | 750 | 1.59 | 0.048 | 1.44 | Lipid metabolism |
| 15 | Hexadecane | 11006 | 6.17 | 0.001 | 1.66 | Lipid metabolism |
| 16 | O-Phosphorylethanolamine | 1015 | 1.57 | ＜0.001 | 1.53 | Lipid metabolism |
| 17 | Glycerol | 753 | 1.21 | 0.035 | 1.22 | Carbohydrate metabolism |
| 18 | Lactic acid | 107689 | 1.32 | 0.013 | 3.11 | Carbohydrate metabolism |
| 19 | Myo-inositol | 892 | 1.31 | 0.021 | 5.7 | Carbohydrate metabolism |
| 20 | Ribose | 5779 | 1.57 | 0.008 | 1.03 | Carbohydrate metabolism |
| 21 | 3-phosphoglycerate | 724 | 1.65 | 0.027 | 1.75 | Carbohydrate metabolism |
| 22 | Phosphate | 1061 | 0.86 | 0.001 | 7.67 | Energy metabolism |
| 23 | Inosine | 6021 | 1.32 | 0.027 | 1.21 | Nucleotide metabolism |
| 24 | Aminooxyacetic acid | 286 | 0.47 | 0.012 | 1.14 | Other |
| 25 | Oxamic acid | 974 | 1.32 | 0.006 | 1 | Other |

VIP, variable importance on projection;

^a^ Fold Change was calculated as the ratio of the average variable value in the CSDS group to that in the CON group.
