# Supplementary material for: Integrated Metabolomics and Proteomics Analysis Revealed Second Messenger System Disturbance in Hippocampus of Chronic Social Defeat Stress Rat
Source: Front Neurosci. 2019 Mar 22;13:247. doi: 10.3389/fnins.2019.00247 (PMC6448023; doi:10.3389/fnins.2019.00247)
Supplement: TABLE S2 — Identified differential proteins in hippocampus between the CSDS and CON groups. [file Table_2.DOCX]

**Table 2.** Identified differential proteins in hippocampus between the CSDS and CON groups.

| **No.** | **UniProt Accession** | **Gene name** | **Protein name** | **Fold change^a^** | | | **p-value** | |
| --- | --- | --- | --- | --- | --- | --- | --- | --- |
| 1 | A0A096MJI4 | Gigyf2 | GRB10 interacting GYF protein 2 | 0.585 | | | 0.008 | |
| 2 | A0A096MJJ6 | Acbd5 | Acyl-CoA-binding domain-containing protein 5 | 0.71 | | | 0.012 | |
| 3 | A0A096MJV2 | Pdzd11 | PDZ domain-containing 11 | 0.791 | | | 0.028 | |
| 4 | A0A096MKD9 | Trim46 | Tripartite motif-containing protein 46 | 0.742 | | | 0.017 | |
| 5 | A0A0A0MXX1 | Akap7 | A-kinase anchor protein 7 isoforms delta and gamma | 1.239 | | | 0.031 | |
| 6 | A0A0G2JSL8 | Adk | Adenosine kinase | 0.82 | | | 0.014 | |
| 7 | A0A0G2JSM7 | Add1 | Adducin 1 (Alpha), isoform CRA_b | 0.762 | | | 0.031 | |
| 8 | A0A0G2JSR0 | Vdac3 | Voltage-dependent anion-selective channel protein 3 | 1.203 | | | 0.033 | |
| 9 | A0A0G2JTW9 | Hbb-b1 | Hemoglobin subunit beta | 1.381 | | | 0.011 | |
| 10 | A0A0G2JU85 | Ddn | Dendrin | 0.718 | | | 0.004 | |
| 11 | A0A0G2JW13 | LOC103694210 | NACHT and WD repeat domain-containing protein 2-like | 0.832 | | | 0.026 | |
| 12 | A0A0G2JWT6 | Pex16 | Peroxisomal biogenesis factor 16 | 0.766 | | | 0.013 | |
| 13 | A0A0G2JYC7 | Hdgfl2 | Hepatoma-derived growth factor-related protein 2 | 0.798 | | | 0.035 | |
| 14 | A0A0G2JYV0 | Brd4 | Bromodomain-containing 4 | 0.655 | | | 0.017 | |
| 15 | A0A0G2JZY3 | Reps1 | RALBP1-associated Eps domain-containing 1 | 0.786 | | | 0.033 | |
| 16 | A0A0G2K0M6 | Rpl22 | 60S ribosomal protein L22 | 1.204 | | | 0.027 | |
| 17 | A0A0G2K0S0 | Phactr3 | Phosphatase and actin regulator | 0.723 | | | 0.024 | |
| 18 | A0A0G2K0W0 | Ggps1 | Geranylgeranyl pyrophosphate synthase 1 | 0.822 | | | 0.015 | |
| 19 | A0A0G2K0W1 | Dab1 | Disabled homolog 1 | 1.321 | | | 0.048 | |
| 20 | A0A0G2K140 | Erc2 | ELKS/RAB6-interacting/CAST family member 2 | 0.571 | | | 0.008 | |
| 21 | A0A0G2K285 | Reps2 | RALBP1 associated Eps domain containing protein 2 | 0.8 | | | 0.013 | |
| 22 | A0A0G2K2L1 | Podxl | Podocalyxin | 0.82 | | | 0.048 | |
| 23 | A0A0G2K2M9 | Srrm2 | Serine/arginine repetitive matrix 2 | 0.772 | | | 0.018 | |
| 24 | A0A0G2K3Y9 | Mkl1 | Megakaryoblastic leukemia (Translocation) 1 (Predicted) | 1.21 | | | 0.016 | |
| 25 | A0A0G2K568 | Crym | Ketimine reductase mu-crystallin | 0.816 | | | 0.011 | |
| 26 | A0A0G2K5E8 | NEWGENE_621351 | Collagen, type I, alpha 2 | 1.473 | | | 0.009 | |
| 27 | A0A0G2K5G2 | LOC103691744 | Cystathionine gamma-lyase | 1.246 | | | 0.003 | |
| 28 | A0A0G2K654 | LOC684681 | Histone cluster 1 H1 family member c | 1.511 | | | 0.019 | |
| 29 | A0A0G2K6A2 | Ptpn9 | Protein tyrosine phosphatase, non-receptor type 9 | 0.827 | | | 0.018 | |
| 30 | A0A0G2K8K9 | Rbmxl1b | RNA binding motif protein, X-linked-like 1B | 0.815 | | | 0.001 | |
| 31 | A0A0G2K913 | Prkag1 | 5'-AMP-activated protein kinase subunit gamma-1 | 0.818 | | | 0.016 | |
| 32 | A0A0G2KAJ5 | LOC103690141 | Coiled-coil domain-containing protein 85A-like | 0.812 | | | 0.027 | |
| 33 | A0A0G2KAW7 | Eif4h | Eukaryotic translation initiation factor 4H | 1.219 | | | 0.044 | |
| 34 | A0A0H2UHL8 | Kazn | Kazrin, periplakin interacting protein | 0.718 | | | 0.023 | |
| 35 | A0A0H2UI11 | Rpl39 | 60S ribosomal protein L39 | 0.742 | | | 0.044 | |
| 36 | A1A5P9 | Magee1 | Melanoma-associated antigen E1 | 0.527 | | | 0.007 | |
| 37 | A1L1K3 | Anapc5 | Anaphase-promoting complex subunit 5 | 0.751 | | | 0.007 | |
| 38 | B0BN15 | Shd | Similar to src homology 2 domain-containing transforming protein D | 0.704 | | | 0.041 | |
| 39 | B1WBZ7 | Gpn1 | GPN-loop GTPase 1 | 0.756 | | | 0.007 | |
| 40 | B2GV05 | Rbm5 | RNA-binding protein 5 | 0.802 | | | 0.014 | |
| 41 | B2GV65 | Higd2a | HIG1 domain family, member 2A | 1.226 | | | 0.002 | |
| 42 | B2GV96 | Ccdc115 | Coiled-coil domain-containing 115 | 0.615 | | | 0.019 | |
| 43 | B2RYI0 | Wdr91 | WD repeat-containing protein 91 | 0.804 | | | 0.002 | |
| 44 | B2RZD1 | Sec61b | Protein transport protein Sec61 subunit beta | 0.816 | | | 0.013 | |
| 45 | B4F786 | Cd2bp2 | CD2 antigen (Cytoplasmic tail) binding protein 2 (Predicted), isoform CRA_a | 0.803 | | | 0.012 | |
| 46 | B5DFK8 | Pdxdc1 | Pdxdc1 protein | | 0.777 | 0.013 | |  |
| 47 | C7EMI4 | N/A | Cytochrome b | | 1.301 | | 0.016 | |

| 48 | C9EH87 | Sptan1 | Spectrin alpha, non-erythrocytic 1 | 1.261 | 0.035 |
| --- | --- | --- | --- | --- | --- |
| 49 | D3Z8Q7 | Fam96b | Family with sequence similarity 96, member B | 0.732 | 0.007 |
| 50 | D3Z956 | Rlbp1 | Retinaldehyde binding protein 1 (Predicted), isoform CRA_a | 0.777 | 0 |
| 51 | D3Z9C0 | Mios | Meiosis regulator for oocyte development | 0.798 | 0.005 |
| 52 | D3ZAS8 | Sart3 | Squamous cell carcinoma antigen recognized by T-cells 3 | 0.811 | 0.044 |
| 53 | D3ZBL6 | Nup160 | Nucleoporin 160 | 0.666 | 0.047 |
| 54 | D3ZC15 | Carmil2 | Capping protein regulator and myosin 1 linker 2 | 0.797 | 0.013 |
| 55 | D3ZCR3 | LOC108349189 | High mobility group protein B1 pseudogene | 1.204 | 0.005 |
| 56 | D3ZE63 | LOC679748 | Protein LOC679748 | 0.822 | 0.033 |
| 57 | D3ZFY7 | Psd3 | Pleckstrin and Sec7 domain-containing 3 | 1.264 | 0.002 |
| 58 | D3ZHP7 | Ulk3 | Serine/threonine-protein kinase ULK3 | 0.826 | 0.013 |
| 59 | D3ZIF0 | Zfp512 | Zinc finger protein 512 | 0.8 | 0.02 |
| 60 | D3ZIP8 | Endod1 | Protein Endod1 | 1.2 | 0.004 |
| 61 | D3ZJ92 | Prpf40a | Pre-mRNA processing factor 40 homolog A (Yeast) (Predicted) | 0.756 | 0.021 |
| 62 | D3ZJD3 | RGD1565183 | protein LOC100362069 | 1.234 | 0 |
| 63 | D3ZJZ1 | Atg5 | Autophagy protein 5 | 0.747 | 0.038 |
| 64 | D3ZKR8 | Tmem167a | Protein kish | 0.827 | 0.023 |
| 65 | D3ZKT0 | Tamm41 | Phosphatidate cytidylyltransferase, mitochondrial | 0.824 | 0.013 |
| 66 | D3ZMK9 | Pragmin | Inactive tyrosine-protein kinase PRAG1 | 0.822 | 0.016 |
| 67 | D3ZPT1 | RGD1562747 | Similar to RIKEN cDNA 1110012L19 | 0.702 | 0.028 |
| 68 | D3ZQE8 | Xpo5 | Exportin 5 | 0.827 | 0.016 |
| 69 | D3ZQI1 | Gpx7 | Glutathione peroxidase | 0.761 | 0.021 |
| 70 | D3ZS15 | N/A | Uncharacterized protein | 0.566 | 0.001 |
| 71 | D3ZTX0 | Tmed7 | Transmembrane emp24 domain-containing protein 7 | 1.299 | 0.038 |
| 72 | D3ZW27 | Map3k5 | Mitogen-activated protein kinase kinase kinase 5 | 0.741 | 0.047 |
| 73 | D3ZXY2 | Pdzd8 | PDZ domain containing 8 (Predicted) | 0.795 | 0.042 |
| 74 | D3ZY71 | LOC100910137 | RCG21156 | 1.214 | 0.023 |
| 75 | D3ZZN4 | LOC100362338 | LOC100362338 | 1.466 | 0.026 |
| 76 | D4A2H8 | Nudt12 | Nudix (Nucleoside diphosphate linked moiety X)-type motif 12 (Predicted) | 1.209 | 0.01 |
| 77 | D4A3L3 | Megf9 | EGF-like-domain, multiple 5 (Predicted) | 1.246 | 0.024 |
| 78 | D4A4P8 | Ttyh2 | Protein tweety homolog | 0.697 | 0.047 |
| 79 | D4A507 | Clip3 | CAP-Gly domain-containing linker protein 3 | 0.659 | 0.002 |
| 80 | D4A520 | Pptc7 | PTC7 protein phosphatase homolog | 0.782 | 0 |
| 81 | D4A5P3 | Fam73a | Mitoguardin 1 | 0.793 | 0.025 |
| 82 | D4A6A8 | Ppp1r13b | Protein phosphatase 1, regulatory (Inhibitor) subunit 13B (Predicted) | 0.796 | 0.034 |
| 83 | D4A709 | Tubgcp6 | Tubulin, gamma complex-associated protein 6 | 0.652 | 0.014 |
| 84 | D4A772 | Dtna | Dystrobrevin | 0.818 | 0.033 |
| 85 | D4A8G0 | Lsm12 | LSM12 homolog | 1.264 | 0.002 |
| 86 | D4AB66 | Ston2 | Stonin-2 | 0.768 | 0.046 |
| 87 | D4ACK1 | Nup214 | Nucleoporin 214 | 0.681 | 0.003 |
| 88 | D4ACM9 | Mfap1a | Inositol hexakisphosphate and diphosphoinositol-pentakisphosphate kinase 1 | 1.209 | 0.031 |
| 89 | D4ACU6 | Adgra1 | Adhesion G protein-coupled receptor A1 | 0.647 | 0.001 |
| 90 | D4ADA1 | Fibcd1 | Fibrinogen C domain containing 1 (Predicted) | 0.687 | 0.013 |
| 91 | E2E1S0 | Cdkl5 | Cyclin-dependent kinase-like 5 | 0.763 | 0.002 |
| 92 | F1LM11 | Nrg2 | Pro-neuregulin-2, membrane-bound isoform | 1.241 | 0.046 |
| 93 | F1LN34 | Ankh | Progressive ankylosis protein homolog | 0.811 | 0.045 |
| 94 | F1LN42 | Tns1 | Tensin 1 | 0.689 | 0.019 |
| 95 | F1LPQ4 | Pld2 | Phospholipase D2 | 0.673 | 0.002 |
| 96 | F1LQ14 | Rpl34 | 60S ribosomal protein L34 | 1.32 | 0.009 |
| 97 | F1LQ27 | Fam98c | Family with sequence similarity 98, member C | 0.81 | 0.041 |
| 98 | F1LQI2 | Robo1 | Roundabout homolog 1 | 0.643 | 0.004 |
| 99 | F1LR84 | Nptx2 | Neuronal pentraxin-2 | 0.698 | 0.013 |
| 100 | F1LRA4 | Grip1 | Glutamate receptor-interacting protein 1 | 0.823 | 0.004 |
| 101 | F1LSB5 | LOC691056 | LOC691056 | 0.824 | 0.017 |
| 102 | F1LV01 | Wdr19 | WD repeat domain 19 | 0.708 | 0.01 |
| 103 | F1LX47 | Iqca1 | IQ and AAA domain-containing protein 1 | 1.361 | 0.001 |
| 104 | F1LZJ4 | Hyi | Putative hydroxypyruvate isomerase | 0.735 | 0.037 |
| 105 | F1M0A0 | Ano3 | Anoctamin | 0.786 | 0.018 |
| 106 | F1M4V6 | Kalrn | Kalirin | 0.74 | 0.028 |
| 107 | F1M6E5 | Nacad | NAC alpha domain-containing | 0.79 | 0.016 |
| 108 | F1M790 | Ptgfrn | Prostaglandin F2 receptor negative regulator | 0.663 | 0.034 |
| 109 | F1M842 | Tp53bp1 | Tumor protein p53-binding protein 1 | 0.795 | 0.001 |
| 110 | F1M8Y4 | Deptor | DEP domain-containing MTOR-interacting protein | 0.715 | 0.002 |
| 111 | F1M9C0 | Mapkapk2 | Mitogen-activated protein kinase-activated protein kinase 2 | 0.509 | 0.049 |
| 112 | F1M9X4 | Clic6 | Chloride intracellular channel protein | 0.695 | 0.013 |
| 113 | F1MAL5 | Irs2 | Insulin receptor substrate 2 | 0.721 | 0.047 |
| 114 | F7F3Z1 | Lman2l | Lectin, mannose-binding 2-like (Predicted), isoform CRA_c | 0.795 | 0.008 |
| 115 | G3V6D9 | Slc9a3r2 | Na(+)/H(+) exchange regulatory cofactor NHE-RF | 0.794 | 0.016 |
| 116 | G3V6R0 | Slc1a2 | Amino acid transporter | 0.484 | 0.016 |
| 117 | G3V6X7 | LOC108348172 | ProSAAS | 0.816 | 0.043 |
| 118 | G3V6Z5 | Gria3 | Glutamate receptor 3 | 1.299 | 0.003 |
| 119 | G3V7Z6 | Abcd2 | ATP-binding cassette sub-family D member 2 | 1.221 | 0.019 |
| 120 | G3V8P3 | Celsr2 | Cadherin EGF LAG seven-pass G-type receptor 2 | 0.821 | 0.014 |
| 121 | I7EFB0 | Mbp | Myelin basic protein transcript variant N | 1.407 | 0.015 |
| 122 | L0N559 | Slc4a5 | Anion exchange protein | 1.23 | 0.046 |
| 123 | M0R6K5 | Adgrv1 | Adhesion G protein-coupled receptor V1 | 0.787 | 0.017 |
| 124 | M0R749 | Tspan15 | Tetraspanin | 1.228 | 0.016 |
| 125 | M0R7B4 | LOC684828 | Histone cluster 1 H1 family member d | 1.294 | 0.004 |
| 126 | M0RAD5 | Clpp | ATP-dependent Clp protease proteolytic subunit | 1.208 | 0.048 |
| 127 | M0RCH4 | N/A | Uncharacterized protein | 0.821 | 0.045 |
| 128 | O08589 | Fxyd1 | Phospholemman | 0.809 | 0.03 |
| 129 | O08871 | Kcnj3 | G-protein coupled inward rectifier potassium channel splice variant Kir3.1b | 1.234 | 0.015 |
| 130 | O35119 | Trpc4 | Short transient receptor potential channel 4 | 0.815 | 0.038 |
| 131 | O35147 | Bad | Bcl2-associated agonist of cell death | 0.82 | 0.019 |
| 132 | O35276 | Nrp2 | Neuropilin-2 | 0.803 | 0.004 |
| 133 | O55145 | Cx3cl1 | Fractalkine | 0.738 | 0.042 |
| 134 | O55215 | Rps2-ps6 | Ribosomal protein S2 | 1.204 | 0.002 |
| 135 | O88267 | Acot1 | Acyl-coenzyme A thioesterase 1 | 0.732 | 0.005 |
| 136 | O88370 | Pip4k2c | Phosphatidylinositol 5-phosphate 4-kinase type-2 gamma | 1.203 | 0.016 |
| 137 | O88778 | Bsn | Protein bassoon | 0.726 | 0.001 |
| 138 | P01041 | Cstb | Cystatin-B | 1.268 | 0.047 |
| 139 | P01355 | Cck | Cholecystokinin | 0.788 | 0.005 |
| 140 | P02696 | Rbp1 | Retinol-binding protein 1 | 0.808 | 0.011 |
| 141 | P04762 | Cat | Catalase | 0.828 | 0.015 |
| 142 | P05714 | Rab4a | Ras-related protein Rab-4A | 1.203 | 0.014 |
| 143 | P0C2C0 | Mrpl22 | 39S ribosomal protein L22, mitochondrial | 0.735 | 0.003 |
| 144 | P0C627 | Ubxn2b | UBX domain-containing protein 2B | 0.822 | 0.033 |
| 145 | P0C644 | Ppip5k1 | Inositol hexakisphosphate and diphosphoinositol-pentakisphosphate kinase 1 | 0.543 | 0.028 |
| 146 | P10818 | Cox6a1 | Cytochrome c oxidase subunit 6A1, mitochondrial | 1.215 | 0.035 |
| 147 | P13668 | Stmn1 | Stathmin | 1.225 | 0.03 |
| 148 | P14841 | Cst3 | Cystatin-C | 1.229 | 0.034 |
| 149 | P18437 | Hmgn2 | Non-histone chromosomal protein HMG-17 | 2.138 | 0.048 |
| 150 | P18508 | Gabrg2 | Gamma-aminobutyric acid receptor subunit gamma-2 | 0.705 | 0.014 |
| 151 | P23565 | Ina | Alpha-internexin | 1.314 | 0.001 |
| 152 | P25122 | Kcnc1 | Potassium voltage-gated channel subfamily C member 1 | 0.808 | 0.032 |
| 153 | P25886 | Rpl29 | 60S ribosomal protein L29 | 1.451 | 0.013 |
| 154 | P26431 | Slc9a1 | Sodium/hydrogen exchanger 1 | 0.831 | 0.003 |
| 155 | P36860 | Ralb | Ras-related protein Ral-B | 1.267 | 0.018 |
| 156 | P36877 | Ppp2r2b | Serine/threonine-protein phosphatase 2A 55 kDa regulatory subunit B beta isoform | 0.718 | 0.039 |
| 157 | P41350 | Cav1 | Caveolin-1 | 1.295 | 0.049 |
| 158 | P41413 | Pcsk5 | Proprotein convertase subtilisin/kexin type 5 | 1.203 | 0.022 |
| 159 | P47875 | Csrp1 | Cysteine and glycine-rich protein 1 | 1.211 | 0.029 |
| 160 | P51869 | Cyp4f4 | Cytochrome P450 4F4 | 0.557 | 0.019 |
| 161 | P52555 | Erp29 | Endoplasmic reticulum resident protein 29 | 0.807 | 0.011 |
| 162 | P61354 | Rpl27 | 60S ribosomal protein L27 | 1.206 | 0.014 |
| 163 | P62255 | Ube2g1 | Ubiquitin-conjugating enzyme E2 G1 | 0.779 | 0.015 |
| 164 | P62845 | Rps15 | 40S ribosomal protein S15 | 1.259 | 0.002 |
| 165 | P63081 | Atp6v0c | V-type proton ATPase 16 kDa proteolipid subunit | 0.818 | 0.022 |
| 166 | P67999 | Rps6kb1 | Ribosomal protein S6 kinase beta-1 | 0.774 | 0.002 |
| 167 | P81795 | Eif2s3 | Eukaryotic translation initiation factor 2 subunit 3, X-linked | 0.735 | 0.042 |
| 168 | Q08326 | Rabif | Guanine nucleotide exchange factor MSS4 | 0.796 | 0.039 |
| 169 | Q0VGK4 | Gdpd1 | Lysophospholipase D GDPD1 | 1.218 | 0.009 |
| 170 | Q32PX2 | Aimp2 | Aminoacyl tRNA synthase complex-interacting multifunctional protein 2 | 0.801 | 0.047 |
| 171 | Q3T1K9 | Nicn1 | Nicolin 1 | 0.71 | 0.012 |
| 172 | Q498C8 | Rer1 | Protein RER1 | 0.719 | 0.023 |
| 173 | Q498T9 | Lrrc8c | Volume-regulated anion channel subunit LRRC8C | 0.833 | 0.02 |
| 174 | Q4KM31 | Limd2 | LIM domain-containing protein 2 | 1.462 | 0.006 |
| 175 | Q4QQT5 | N/A | Probable isocitrate dehydrogenase [NAD] gamma 2, mitochondrial | 0.811 | 0.019 |
| 176 | Q4V8H8 | Ehd2 | EH domain-containing protein 2 | 0.797 | 0.01 |
| 177 | Q562C9 | Adi1 | 1,2-dihydroxy-3-keto-5-methylthiopentene dioxygenase | 0.734 | 0.001 |
| 178 | Q5BJS9 | Morn4 | MORN repeat-containing protein 4 | 0.771 | 0.013 |
| 179 | Q5FVL9 | Fam103a1 | Fam103a1 protein | 1.247 | 0.027 |
| 180 | Q5FVQ8 | Nlrx1 | NLR family member X1 | 0.738 | 0.011 |
| 181 | Q5PPJ4 | Dohh | Deoxyhypusine hydroxylase | 0.812 | 0.004 |
| 182 | Q5PPN5 | Tppp3 | Tubulin polymerization-promoting protein family member 3 | 1.202 | 0.004 |
| 183 | Q5PQJ6 | Pycr3 | Pyrroline-5-carboxylate reductase 3 | 0.83 | 0.013 |
| 184 | Q5PQN9 | Mrpl38 | 39S ribosomal protein L38, mitochondrial | 0.781 | 0.026 |
| 185 | Q5U201 | Amn1 | Protein AMN1 homolog | 0.629 | 0.015 |
| 186 | Q5U2R3 | Frmd8 | FERM domain-containing protein 8 | 0.72 | 0.027 |
| 187 | Q5U3Z0 | Cfap298 | Cilia- and flagella-associated protein 298 | 0.794 | 0.044 |
| 188 | Q5XI79 | Ndufaf7 | Protein arginine methyltransferase NDUFAF7, mitochondrial | 0.782 | 0.012 |
| 189 | Q5XID0 | Yipf5 | Protein YIPF5 | 0.664 | 0.003 |
| 190 | Q62806 | Znf148 | Zinc finger protein 148 | 0.79 | 0.019 |
| 191 | Q63016 | Slc7a5 | Large neutral amino acids transporter small subunit 1 | 0.819 | 0.031 |
| 192 | Q63186 | Eif2b4 | Translation initiation factor eIF-2B subunit delta | 0.661 | 0.049 |
| 193 | Q63259 | Ptprn | Receptor-type tyrosine-protein phosphatase-like N | 0.81 | 0.045 |
| 194 | Q63327 | Mobp | Myelin-associated oligodendrocyte basic protein | 1.446 | 0.004 |
| 195 | Q63524 | Tmed2 | Transmembrane emp24 domain-containing protein 2 | 1.33 | 0.04 |
| 196 | Q63788 | Pik3r2 | Phosphatidylinositol 3-kinase regulatory subunit beta | 0.736 | 0.042 |
| 197 | Q641X9 | Mrpl9 | 39S ribosomal protein L9, mitochondrial | 0.743 | 0.004 |
| 198 | Q641Z8 | Pef1 | Peflin | 0.83 | 0.043 |
| 199 | Q64259 | Vhl | von Hippel-Lindau disease tumor suppressor | 0.707 | 0.025 |
| 200 | Q642B5 | Armcx2 | Armadillo repeat-containing, X-linked 2 | 0.759 | 0.004 |
| 201 | Q66H91 | Git2 | G protein-coupled receptor kinase interacting ArfGAP 2 | 0.672 | 0.026 |
| 202 | Q66HF7 | Pja1 | LOC683077 protein | 0.712 | 0.01 |
| 203 | Q66HG8 | Ik | Protein Red | 0.698 | 0.033 |
| 204 | Q68FY1 | Nup35 | Nucleoporin NUP35 | 0.707 | 0.018 |
| 205 | Q68G39 | Cdk16 | Cyclin-dependent kinase 16 | 0.775 | 0.048 |
| 206 | Q6AYA4 | Galnt14 | Polypeptide N-acetylgalactosaminyltransferase | 0.637 | 0.039 |
| 207 | Q6IN37 | Gm2a | GM2 ganglioside activator | 0.777 | 0.03 |
| 208 | Q6IRI8 | Smad5 | Smad5 protein | 0.656 | 0.044 |
| 209 | Q6MGA9 | Brd2 | Bromodomain-containing protein 2 | 0.804 | 0.007 |
| 210 | Q6P7S0 | Pkm | Pyruvate kinase | 1.247 | 0.041 |
| 211 | Q6PCT8 | Sdhd | Succinate dehydrogenase [ubiquinone] cytochrome b small subunit, mitochondrial | 1.216 | 0.006 |
| 212 | Q6RUV5 | Rac1 | Ras-related C3 botulinum toxin substrate 1 | 1.209 | 0.031 |
| 213 | Q765A7 | Pgap1 | GPI inositol-deacylase | 1.265 | 0.041 |
| 214 | Q7M0B5 | N/A | Protein-tyrosine kinase IIB, 38K | 1.236 | 0.025 |
| 215 | Q7TP07 | Vps13a | Da1-12 | 0.785 | 0.032 |
| 216 | Q7TP42 | Sec62 | Ab2-292 | 1.207 | 0.005 |
| 217 | Q7TQN4 | Rela | RELA | 0.71 | 0.005 |
| 218 | Q80XF7 | Gjc2 | Gap junction gamma-2 protein | 0.709 | 0.04 |
| 219 | Q8CFC9 | Lzts1 | Leucine zipper putative tumor suppressor 1 | 0.795 | 0.003 |
| 220 | Q8R553 | Clstn3 | Calsyntenin-3 | 0.525 | 0.027 |
| 221 | Q8VIF7 | Selenbp1 | Methanethiol oxidase | 0.831 | 0.021 |
| 222 | Q91XQ2 | Epm2a | Laforin | 0.762 | 0.013 |
| 223 | Q920H8 | Heph | Hephaestin | 0.815 | 0.008 |
| 224 | Q920J3 | Coro6 | Coronin-6 | 0.662 | 0.023 |
| 225 | Q924Y3 | Cldn11 | Claudin | 1.288 | 0.006 |
| 226 | Q9ERM7 | Scamp2 | Secretory carrier-associated membrane protein | 0.52 | 0.025 |
| 227 | Q9JHB5 | Tsnax | Translin-associated protein X | 0.816 | 0.032 |
| 228 | Q9JHM3 | RT1 | MHC class I antigen | 0.704 | 0.034 |
| 229 | Q9JHY2 | Sfxn3 | Sideroflexin-3 | 1.52 | 0.015 |
| 230 | Q9JIR4 | Rims1 | Regulating synaptic membrane exocytosis protein 1 | 0.806 | 0.041 |
| 231 | Q9JJW1 | Tspan2 | Tetraspanin-2 | 1.283 | 0.038 |
| 232 | Q9JKM5 | S1pr5 | Sphingosine 1-phosphate receptor 5 | 0.69 | 0.022 |
| 233 | Q9WU61 | Clcc1 | Chloride channel CLIC-like protein 1 | 0.795 | 0.009 |
| 234 | Q9Z2T2 | Kcnk1 | Potassium channel subfamily K member 1 | 0.818 | 0.021 |

^a^ Fold Change was calculated as the ratio of the average variable value in the CSDS group to that in the CON group.
