# Supplementary material for: Integrated Metabolomics and Proteomics Analysis Revealed Second Messenger System Disturbance in Hippocampus of Chronic Social Defeat Stress Rat
Source: Front Neurosci. 2019 Mar 22;13:247. doi: 10.3389/fnins.2019.00247 (PMC6448023; doi:10.3389/fnins.2019.00247)
Supplement: TABLE S3 — Top ten canonical pathways by IPA analysis of integrated data from proteomics and metabolomics. [file Table_3.DOCX]

**Table 3.** Top ten canonical pathways by IPA analysis of integrated data from proteomics and metabolomics.

| **No.** | **Canonical pathways** | **p-value** | **Proteins or Metabolites** |
| --- | --- | --- | --- |
| 1 | Rac Signaling | 3.26×10^-5^ | Ank1, Irs2, Pik3r2, Pip4k2c, Rac1, Ralb, Rela, Rps6kb1 |
| 2 | Ceramide Signaling | 6.63×10^-5^ | Bad, Irs2, Pik3r2, Ppp2r2b, Ralb, Rela, S1pr5 |
| 3 | Folate Transformation I | 1.47×10^-4^ | Glycine, L-methionine, L-serine, Phosphate |
| 4 | S-methyl-5-thio-a-D-ribose 1-phosphate Degradation | 1.86×10^-4^ | Adl1, L-methionine, Phosphate |
| 5 | Regulation of eIF4 and p70S6K Signaling | 2.34×10^-4^ | Eif2b4, Eif2s3, Irs2, Pik3r2, Ppp2r2b, Ralb, Rps15, Rps6kb1 |
| 6 | mTOR Signaling | 2.46×10^-4^ | Irs2, Pik3r2, Pld2, Ppp2r2b, Prkag1, Rac1, Ralb, Rps15, Rps6kb1 |
| 7 | 3-phosphoinositide Biosynthesis | 2.55×10^-4^ | Irs2, Phosphate, Nudt12, Pik3r2, Pip4k2c, Ppip5k1, Ppp1r13b, Pptc7, Ptprn |
| 8 | LPS-stimulated MAPK Signaling | 2.94×10^-4^ | Irs2, Map3k5, Rac1, Ralb, Rela |
| 9 | NGF Signaling | 2.97×10^-4^ | Irs2, Map3k5, Pik3r2, Rac1, Ralb, Rela, Rps6kb1 |
| 10 | PI3K/AKT Signaling | 3.73×10^-4^ | Bad, Map3k5, Pik3r2, Ppp2r2b, Ralb, Rela, Rps6kb1 |
